# Subsidized optimal ART for HIV-positive temporary residents of Australia improves virological outcomes: results from the Australian HIV Observational Database Temporary Residents Access Study

**DOI:** 10.7448/IAS.18.1.19392

**Published:** 2015-02-12

**Authors:** Kathy Petoumenos, Jo Watson, Bill Whittaker, Jennifer Hoy, Don Smith, Lisa Bastian, Robert Finlayson, Andrew Sloane, Stephen T. Wright, Hamish McManus, Matthew G Law

**Affiliations:** 1 The Kirby Institute, UNSW Australia, Sydney, Australia; 2 National Association of People with HIV Australia (NAPWHA), Sydney, Australia; 3 Department of Infectious Diseases, The Alfred Hospital and Monash University, Melbourne, Australia; 4 Albion Centre, Sydney, Australia; 5 School of Public Health and Community Medicine, UNSW, Sydney, Australia; 6 Western Australian Department of Health, Perth, Australia; 7 Taylor Square Private Clinic, Sydney, Australia; 8 AbbVie Pty Ltd., Sydney, Australia

**Keywords:** antiretroviral therapy, treatment access, temporary residents, HIV-positive

## Abstract

**Introduction:**

HIV-positive (HIV+) temporary residents living in Australia legally are unable to access government subsidized antiretroviral treatment (ART) which is provided via Medicare to Australian citizens and permanent residents. Currently, there is no information systematically being collected on non-Medicare eligible HIV+ patients in Australia. The objectives of this study are to describe the population recruited to the Australian HIV Observational Database (AHOD) Temporary Residents Access Study (ATRAS) and to determine the short- and long-term outcomes of receiving (subsidized) optimal ART and the impact on onwards HIV transmission.

**Methods:**

ATRAS was established in 2011. Eligible patients were recruited via the AHOD network. Key HIV-related characteristics were recorded at baseline and prospectively. Additional visa-related information was also recorded at baseline, and updated annually. Descriptive statistics were used to describe the ATRAS cohort in terms of visa status by key demographic characteristics, including sex, region of birth, and HIV disease status. CD4 cell count (mean and SD) and the proportion with undetectable (<50 copies/ml) HIV viral load are reported at baseline, 6 and 12 months of follow-up. We also estimate the proportion reduction of onward HIV transmission based on the reduction in proportion of people with detectable HIV viral load.

**Results:**

A total of 180 patients were recruited to ATRAS by June 2012, and by July 2013 39 patients no longer required ART via ATRAS, 35 of whom became eligible for Medicare-funded medication. At enrolment, 63% of ATRAS patients were receiving ART from alternative sources, 47% had an undetectable HIV viral load (<50 copies/ml) and the median CD4 cell count was 343 cells/µl (IQR: 222–479). At 12 months of follow-up, 85% had an undetectable viral load. We estimated a 75% reduction in the risk of onward HIV transmission with the improved rate of undetectable viral load.

**Conclusions:**

The immunological and virological improvements highlight the importance of supplying optimal ART to this vulnerable population. The increase in proportion with undetectable HIV viral load shows the potentially significant impact on HIV transmission in addition to the personal health benefit for each individual.

## Introduction

The Australian government provides fully subsidized antiretroviral treatment (ART) through the Pharmaceutical Benefits Scheme (PBS) Section 100 (s100) Highly Specialized Drugs programme. To receive ART under this scheme, a patient has to be entitled to a Medicare card. Temporary residents in Australia, under various visa arrangements, are not eligible for a Medicare card and hence cannot currently access fully subsidized ART. These visas include Student visa (international students allowed in Australia to study for the duration of their degree), Working visa (often a professional or employer-sponsored Working visa allowing people from overseas to work full time and earn an income in Australia for a fixed duration of up to four years) and Spousal visas (person from overseas married to an Australian citizen). Other visas such as Bridging visa are often used as individuals are transitioning from one visa type to another. People visiting Australia under most of these visas are expected to cover their health costs through private health insurance which will cover doctor's visits, pathology costs and prescription drugs in the general PBS but not the s100 Highly Specialized Drugs programme.

Although living in Australia legally, HIV-positive temporary residents are not entitled to the same level of care as HIV-positive permanent residents. According to a 2007 survey of s100 prescribing general practitioners, of their HIV-positive temporary resident caseload, only 60% of patients who should be on ART were receiving effective ART, while 31% were said to be receiving sub-optimal treatment; these include regimens or individual ARVs that are no longer considered as recommended or standard of care in Australia [1]. The ART regimens were limited to what was available in the countries they sourced their ART from and not necessarily what is considered optimal and current standard treatment in Australia. Due to their Medicare ineligibility, HIV-positive temporary residents in most instances must obtain their ART medications by paying for their treatment and often at full cost, with no provision for subsidized arrangements through the s100 Highly Specialized Drugs PBS. Cost of treatment is prohibitive for most individuals, particularly if purchased within Australia. For example, Atripla, a co-formulated tablet containing three antiretrovirals, and currently one of the recommended first line regimens, is estimated to cost AUD$12,440 per individual annually in Australia [2].

The majority of HIV-positive temporary residents are thought to source their ART from their country of origin, or overseas online, and most are in generic form. A smaller proportion receive ART by participating in Australian clinical trials while a few pay full price or receive ART via various individual compassionate access requests to some pharmaceutical companies [1,3,4]. Accessing ART overseas poses serious issues for clinicians prescribing ART. Costs influence drug choice and subsequently may prevent appropriate treatment for the individual. Many antiretroviral drugs currently considered optimal standard treatment are not available in generic form, while some are not available in countries where these patients may have to return to live. Ordering overseas may also result in treatment interruptions due to late ordering or stock supply issues leading to additional and frequent unscheduled treatment visits.

HIV diagnoses are increasing in Australia. A key focus of the current national HIV strategy (Sixth National HIV Strategy 2010–2013) is reducing the rate of HIV transmission, along with minimizing the personal and social impacts of HIV infection [5]. Aiming for universal ART coverage, therefore, has significant public health implications, in particular, reducing risk of HIV transmission.

To date there are no systematic data collected on this population. It remains unclear what proportion of this population require ART (i.e. meet Australian guidelines for commencing ART); what level of ART they are currently receiving; what stage of their HIV disease they are in; their age, gender, country of origin, length of time in the country; and whether they obtain permanent residency or whether they return home. Furthermore, the impact of sub-optimal treatment and care for this group on their long-term disease outcome is not well understood or described.

The objective of this paper is to describe the population of HIV-positive temporary resident patients who are currently ineligible for subsidized ART via the s100 scheme in the Australian HIV Observational Database (AHOD) Temporary Residents Access Study (ATRAS), in particular, to describe the HIV disease status of these patients and the short- and long-term outcomes of receiving optimal ART and finally to provide preliminary estimates of risk of onwards HIV transmission in Australia if HIV-positive temporary residents do not receive effective ART.

## Methods

### Establishment of ATRAS

During 2010 and 2011, the National Association of People with HIV Australia (NAPWHA), the peak body for people living with HIV, engaged pharmaceutical companies with registered HIV antiretroviral drugs in Australia to commit to providing ART to 180 HIV-positive temporary residents in Australia for up to four years. By July 2011, all seven companies (AbbVie Pty Ltd., Boehringer-Ingelheim Pty Ltd., Bristol-Myers Squibb Australia Pty Ltd., Gilead Sciences Pty Ltd., Janssen-Cilag Pty Ltd., MSD Pty Ltd. and ViiV Healthcare Pty Ltd.) had committed to this scheme. The ATRAS commenced in November 2011.

### Inclusion criteria

HIV-positive patients who are currently under clinical care and temporary residents who are ineligible for Medicare or for any other programme that can provide ART access, and who satisfied a low-income threshold as set by Centrelink Low Income Health Care Card or deemed by the treating clinician as unable to afford treatment, were eligible to participate in ATRAS. Clinicians approached all HIV-positive patients who met these criteria and invited them to participate in the study. Patients were recruited via the AHOD, a long-term prospective observational cohort study of more than 3000 HIV-positive patients. AHOD commenced in 1999 and is a collaboration of 29 tertiary referral centres, sexual health clinics and specialist general practices throughout most states and territories of Australia [6]. Recruitment was capped at 180 patients and was competitive across sites.

Recruitment via AHOD allowed a standardized patient follow-up and monitoring mechanism. The core data variables collected in AHOD include clinical and treatment information recorded in most HIV treatment clinics as part of routine clinic care. AHOD is an entirely observational study; patients therefore are not required to make any additional visits or undergo any additional tests other than those dictated by local standard of care. These data are electronically transferred to The Kirby Institute, UNSW Australia, where AHOD is managed.

### Data collection

The HIV-related variables routinely collected in AHOD have previously been described in detail [6]. Data for AHOD are collected every six months on a core set of demographic and clinical variables, including sex, age, HIV exposure, hepatitis B virus (HBV) surface antigen, hepatitis C virus (HCV) antibody, CD4 and CD8 cell counts, plasma HIV viral load, ART history, AIDS illnesses and date and cause of death. Data are transferred electronically to The Kirby Institute and are subjected to quality control and quality assurance procedures.

For ATRAS patients, the following data variables are also collected at the time of enrolment: visa status and type, country of origin, year arrived in Australia and employment status. In addition to the regular six monthly clinical data transfers, there is an annual update for ATRAS patients regarding visa status, employment status, if they have applied for permanent residency or if they have become eligible for Medicare.

Ethics approval for AHOD and ATRAS is granted by the UNSW Australia Human Research Ethics Committee and from ethics committees with local jurisdiction over participating sites as required. All study procedures were developed in accordance with the revised 1975 Helsinki Declaration. All participants (AHOD and ATRAS) were required to provide written informed consent prior to enrolment. Strict procedures for maintaining patient confidentiality were adhered to at all times.

### Analyses

Descriptive summary statistics (*n*, mean, standard deviations [SD], median and minimum maximum) or frequency counts and proportions of baseline data are presented. Baseline patient characteristics are described by visa-related characteristics stratified by patient demographics and HIV-related characteristics, including visa type, country of origin, prior ART history and HIV disease stage as defined by CD4 cell count, viral load and AIDS diagnosis.

Changes in CD4 count, in proportion with undetectable viral load (≤50 copies/ml) from baseline to 6 and 12 months of follow-up, crude rates for coming off ATRAS-supplied ART and reasons for no longer requiring ART via ATRAS (e.g. have become Medicare eligible, or have returned home/left the country) are summarized.

The risk of onwards HIV transmission following commencement of ART via ATRAS was determined using two different methods. First, the reduction in proportion with detectable viral load at 12 months from baseline as a direct estimate of the reduction in the risk of onwards HIV transmission. Second, a more quantitative estimate of reduction in the risk of transmission using the method published by Wilson *et al*. [7]. This method is based on the Rakai study of HIV transmission in heterosexual couples, in which each 10-fold reduction in HIV viral load was associated with a 2.45-fold reduction in the risk of transmission. Both methods assumed that the commencement of ART does not affect sexual or other HIV-transmission risk behaviours [8].

## Results

Recruitment to ATRAS commenced on 7 November, 2011, and was completed by end of June 2012 when a total of 180 patients ineligible for Medicare were enrolled from 21 AHOD sites. Details are summarized in Table 1. The majority of ATRAS patients were male (*N*=133; 74%). The mean (SD) age for men and women was similar, 35.2 (9.40) and 35.0 (6.77), respectively. Most of the participants were recruited via sexual health clinics (*N*=82, 46%) followed evenly by general practice (27%) and tertiary referral centres (27%). Most men were recruited from general practices (GP: 34%) or sexual health clinics (44%). The majority of women were recruited via tertiary referral centres (41%) or sexual health clinics (50%), with only a few women recruited via GPs (9%).

**Table 1:** Patient baseline characteristics

	Female		Male		Total	
		
	*N*	%	*N*	%	*N*	%
Total	47		133		180	
Mean age (SD)	35.0	(6.77)	35.2	(9.40)	35.1	(8.77)
AHOD clinic type						
General practice	5	10.6	44	33.1	49	27.2
Tertiary referral centre	19	40.4	30	22.6	49	27.2
Sexual health clinic	23	48.9	59	44.4	82	45.6
Visa type						
Bridging	2	4.3	24	18.0	26	14.4
Other	11	23.4	12	9.0	23	12.8
Spouse	10	21.3	6	4.5	16	8.9
Student	15	31.9	45	33.8	60	33.3
Working	9	19.1	46	34.6	55	30.6
Region						
Asia/SE Asia	21	44.7	61	45.9	82	45.6
Europe	0	0.0	16	12.0	16	8.9
North America	1	2.1	9	6.8	10	5.6
South America	1	2.1	18	13.5	19	10.6
South pacific	9	19.1	10	7.5	19	10.6
Sub-Saharan Africa	15	31.9	19	14.3	34	18.9
World bank criteria						
High income	2	4.3	33	24.8	35	19.4
Upper-middle income	17	36.2	58	43.6	75	41.7
Lower-middle income	20	42.6	30	22.6	50	27.8
Low income	8	17.0	12	9.0	20	11.1
HIV exposure category						
MSM (+MSM/IDU)	0	0.0	89	66.9	89	49.4
Heterosexual	40	85.1	30	22.6	70	38.9
Other/missing	7	14.9	14	10.5	21	11.7
Baseline CD4 cells/µl						
<200	8	17.0	22	16.5	30	16.7
≥200 and <350	12	25.5	41	30.8	53	29.4
≥350	23	48.9	56	42.1	79	43.9
Missing	4	8.5	14	10.5	18	10.0
Mean (SD)	349	(185)	378	(238)	370	(225)
Median (IQR)	360	(238–470)	340	(220–480)	343	(222–479)
HIV viral load						
Undetectable (≤50 copies/ml)	21	44.7	54	40.6	75	41.7
Detectable	21	44.7	63	47.4	84	46.7
Missing	5	10.6	16	12.0	21	11.7
Mean (SD)	122,043	(600,178)	57,357	(135,360)	74,444	(328,243)
Median (IQR)	60	(40–2607)	150	(40–67,353)	85	(40–4290)
No prior ART	12	25.5	55	41.4	67	37.2
Prior ART	35	74.5	78	58.6	113	62.8
ART source						
Compassionate access	12	34.3	13	16.7	25	22.1
Country	17	48.6	36	46.2	53	46.9
Full paying	1	2.9	0	0.0	1	0.9
Trial	2	5.7	11	14.1	13	11.5
Other/unknown	3	14.3	18	35.9	21	29.2

The most common visa types were Student visa (33%), closely followed by Working visa (31%) and Bridging visa (14%). The remaining patients were either on Spousal visa (13%) or Other visas (13%). The type of visa varied by sex (Table 1), with similar proportions of men on either Working or Student visas (35 and 34% each), and a further 18% on Bridging visa. Only 4.5% of men were on a Spousal visa. Among females, 19, 23 and 32% were on Working, Spousal and Student visas, respectively, and only 5% were on Bridging visas.

ATRAS patients were from various regions around the world (Table 1). The majority were from Asia/South East Asia (46%), followed by Sub-Saharan Africa (19%), 11% each from South America and South Pacific, 9% from Europe and 6% were from North America. The majority of patients were from Thailand (16% of men and 26% of women). The next most common countries or origin were India and Zimbabwe for men (9 and 7%, respectively), and PNG and Zimbabwe for women (13% each).

### HIV-related characteristics

The main mode of reported HIV exposure among men was sexual exposure from men who have sex with men (66%) followed by heterosexual contact (23%). Among women, the majority reported heterosexual contact (85%). Less than 2% of the ATRAS patients reported injecting drug use as mode of HIV exposure (Table 1). At the time of enrolment, 63% of patients were already receiving ART, slightly greater proportions of women (74%) compared to men (59%). The main source of ART was from overseas (47%), compassionate access (22%) or clinical trial (11%). Of those who received prior ART, twice as many women (34%) compared with men (17%) received ART via compassionate access.

Among patients with a CD4 cell count recorded within one year prior to enrolment (*N*=162), the median (IQR) CD4 count was 343 cells/µl (222–479). Median CD4 count was similar for men and women, but somewhat lower among participants not previously receiving ART (285 cells/µl; IQR: 216–350) compared to those receiving ART (384 cells/µl; IQR: 238–520). Among patients with a HIV viral load measure available within one year prior to enrolment into ATRAS (*N*=159), 47% had undetectable (<50 copies/ml) viral loads. Of patients receiving ART prior to enrolment, 72% had undetectable viral loads compared to only 2% of patients not on treatment. Approximately 70% of patients who had a baseline CD4 count above 350 cells/µl had undetectable viral loads, compared to 25% or less for the lower CD4 categories. Regional and visa differences are also observed and reported in Table 2.

**Table 2:** Undetectable HIV viral load (<50 copies/ml) at baseline, 6 and 12 months

	Baseline		6 months		12 months	
		
	(*N*=159)^a^		(*N*=138)^b^		(*N*=120)^c^	
		
	*N*	%	*N*	%	*N*	%
Total	75	47.2	120	87	106	88.3
Female	21	50	29	80.6	26	81.3
Male	54	46.2	91	89.2	80	90.9
Visa type						
Bridging	14	60.9	16	94.1	19	86.4
Other	12	60	16	88.9	13	81.3
Spouse	6	40	10	71.4	7	77.8
Student	21	38.9	39	86.7	33	97.1
Working	22	46.8	39	88.6	34	87.2
Region						
Asia/SE Asia	31	41.9	57	85.1	47	90.4
Europe	7	50	13	100	13	100
North America	5	62.5	6	85.7	4	66.7
South America	5	27.8	13	100	13	100
South Pacific	8	57.1	8	72.7	10	76.9
Sub-Saharan Africa	19	61.3	23	85.2	19	82.6
Prior ART	74	71.8	79	94	71	91
Baseline CD4 (cells/µl)						
<200	7	24.1	19	76	16	80
≥200 and<350	13	25.5	41	87.2	31	88.6
≥350	55	70.5	53	91.4	51	89.5
Missing	0	0	7	87.5	8	100

a Number with HIV viral load measure within 12 months prior to enrolment

b number with HIV viral load measure within 2 months of a 6- month window

c number with HIV viral load measure within 3 months of a 12-month window.

Compared to the overall AHOD population, at enrolment, ATRAS patients were younger (mean age 35 years; SD: 9) than the AHOD patients (mean 42 years; SD: 10), a greater proportion reported heterosexual contact as mode of HIV transmission (39% in ATRAS compared to 17% in AHOD), and included a greater proportion females (26%in ATRAS vs. 8% in AHOD). Mean CD4 count at enrolment was lower in ATRAS (370 [SD: 225] vs. 504 [SD: 281] in AHOD). Proportions with undetectable viral load were also lower among ATRAS patients compared with AHOD patients (ATRAS 47% vs. 60% AHOD).

### Patient outcomes

ATRAS patients were followed for a median of 1.60 years (IQR: 1.34–1.62). There was a marked increase in the proportion of patients with a viral load measure available who had an undetectable (<50 copies/ml) result, 87 and 88% at 6 and 12 months, respectively. For almost all patient characteristics, gender, region, baseline CD4 category, prior ART therapy and visa type, more than 75% had undetectable viral loads by month 12 (Table 2).

Among patients with a baseline CD4 measure and a follow-up CD4 measure at six months (*N*=133), the mean increase in CD4 was 87 cells/µl (SD: 138). Greater mean increases were observed among men (99 cells/µl; SD: 139) compared to women (54 cells/µl; SD: 132), and among patients with lower baseline CD4 counts (CD4<200: 103 cells/µl; SD: 90, and CD4 ≥200–<350: 118 cells/µl; SD: 147) compared to higher baseline CD4 counts ≥350 cells/µl (58 cells/µl; SD: 143). Greater mean increases were also observed among patients who were not on ART at enrolment into ATRAS (120 cells/µl; SD: 112) compared to patients already receiving ART (68 cells/µl; SD: 149) (Table 3). By 12 months, the mean increase in CD4 overall was 119 cells/µl (SD: 165). Similar mean increases were observed for men and women (123 cells/µl [SD: 167] and 110 cells/µl [SD 160], respectively), and greater increases among the lower baseline CD4 cell strata. By month 12, the South Pacific region and North America demonstrated larger mean increases in CD4 change approaching that of the other regions (Table 3).

**Table 3:** Mean change in CD4 cell count at 6 and 12 months of follow-up

	6 months		12 months	
	
	Mean	SD	Mean	SD
Total^a^	87	138	119	165
Female	54	132	110	160
Male	99	139	123	167
Baseline CD4 (cells/µl)				
<200	103	90	143	105
≥200 and<350	118	147	171	157
≥350	58	143	78	177
Visa type				
Bridging	79	200	84	227
Other	49	74	158	104
Spouse	58	107	93	132
Student	115	134	142	150
Working	85	136	106	167
Region				
Asia/SE Asia	90	150	122	176
Europe	104	99	138	170
North America	15	189	99	133
South America	160	143	174	131
South Pacific	36	107	119	131
Sub-Saharan Africa	71	104	76	177
No prior ART	120	112	187	115
Prior ART	68	149	87	176

a Includes only patients with baseline and follow-up measures.

### Rate coming off ATRAS-supplied ART

Since enrolment up to the time of these data analyses (July 2013), 39 patients (31; 23% of males, and 8; 17% of females) were no longer receiving ART via ATRAS, over a total of 179 person years of follow-up. The majority (*N*=33, 85%) had become eligible for Medicare, four had left the country, and two were lost to follow-up.

**Figure 1:**
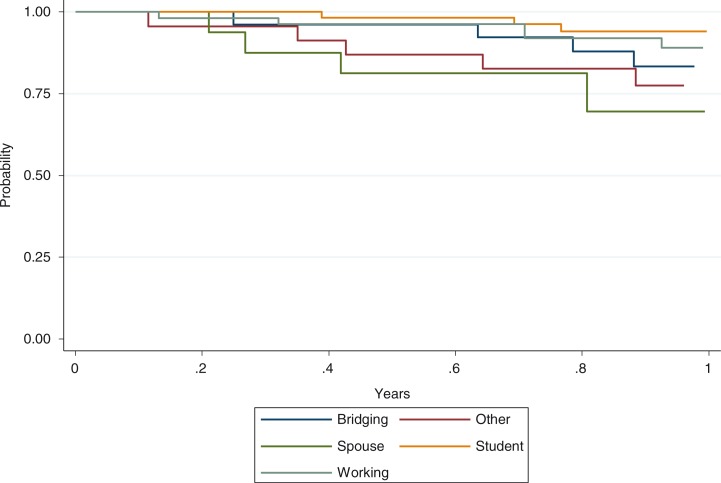
Time for coming off ATRAS-supplied ART.

The overall rate of coming off ATRAS-supplied ART per 100 person years is greatest among patients on Spousal visa (40.1, 95%CI: 16.7–96.4), followed by Bridging (30.8, 95% CI: 16–59.1) and Others (29.7, 95% CI: 14.2–62.4) (Figure 1).

### HIV transmission

Among patients with a baseline viral load measure available, 53% of patients had a detectable HIV viral load. This decreased to 12% at 12 months of follow-up. Assuming that ART does not affect sexual or other HIV-transmission risk behaviours, this represents a 77.4% reduction in the number of patients who have detectable viral load and who have a substantial risk of onward transmission. The mean HIV viral load at baseline was 74,444 copies/ml and 2060 copies/ml at 12 months’ follow-up. Applying the method by Wilson *et al*. [7], and again assuming that ART does not affect sexual or other HIV-transmission risk behaviours, these reductions in mean viral load are estimated to reduce the risk of onwards transmission by 75.2%.

## Discussion

This is the first comprehensive study of HIV-positive Medicare ineligible individuals in Australia. A total of 180 patients were recruited to ATRAS. Although the majority of ATRAS patients were male, 26% were female, which is a larger proportion than that seen in the overall Australian HIV epidemic [9]. More than 60% had received ART at the time of enrolment, yet overall less than half had an undetectable viral load. Most patients sourced their ART from overseas or via compassionate access, while only one patient reportedly paid for the ART at full cost.

Within one year of follow-up in ATRAS and with continued ART supply, the proportion of ATRAS patients with undetectable viral load had increased to 88%, and CD4 cell count increased on average by 119 cells/µl. HIV viral load at 6–12 months highlight a significant potential impact on mediating the risk of onwards HIV transmission. In this analysis we estimated a 75% reduction in potential onward transmission. For the “test and treat” approach towards the elimination of HIV to be successful, which is the cornerstone of the current and future HIV national strategy in Australia, HIV-positive temporary residents must also be included in the treatment model.

During this period, 39 HIV-positive individuals enrolled in ATRAS no longer required ART via the ATRAS mechanism. Four had returned to their home country and 33 individuals had become eligible for Medicare reimbursement (the remaining 2 were lost to follow-up). The overall rate of coming off ATRAS-supplied ART was 22/100 person years. Rates varied by visa type, which broadly reflect the length and type of visa. Student and Working visas are up to four years or so, while the other visa types such as spousal and bridging visas may be shorter in length and approved more quickly. With further follow-up of ATRAS patients we will be able to have a better understanding of differences in these rates by visa type.

Although this is the first standardized study of this population in Australia, there are some limitations. First, the representativeness of the ATRAS population is difficult to determine. ATRAS participants are temporary residents who are currently seeking care through the Australian Health system; however, the exact number of temporary residents who were HIV-positive currently in Australia is unknown. The low number of females in ATRAS also makes it difficult to draw any conclusions regarding sex comparisons, although there are proportionally more females in ATRAS than in AHOD or the general HIV population in Australia. It is also unknown whether their infection was acquired in Australia or prior to entry. The likely place of HIV acquisition has only recently been collected by jurisdictions and therefore we are uncertain how representative this population is of the entire HIV-positive temporary resident population in Australia. HIV testing is not required prior to entry for most people, although it is required for an application for permanent residency. The AHOD group conducted a survey during June and July 2013 of 42 HIV treatment clinics across most states and territories of Australia. From this survey the authors estimated around 450 known HIV-positive patients as temporary Australian residents (unpublished). Although not all HIV treatment clinics were surveyed, exact numbers are not known, the sites surveyed provide healthcare to approximately 70% of the national HIV-positive patient caseload. In terms of estimates of potential HIV transmission, no behavioural or partner HIV status data was available among ATRAS patients. We estimated the effect of the ATRAS study on the risk of onward HIV transmission assuming no change in sexual risk behaviour once on treatment. Whether this is a correct assumption is difficult to ascertain. A meta-analysis in 2004 reported that change in sexual risk behaviour depended largely on the belief of whether being on treatment and with an undetectable viral load decreases the risk of onwards HIV transmission [8], In the current context of “test and treat” starting ART may increase the likelihood of unsafe sex, yet whether this will be the case, and the extent to which is might counterbalance the beneficial effect of treatment on HIV transmission, is currently uncertain, both in this Medicare ineligible population, and the broader HIV-positive population. Further follow-up and data collection on sexual risk behaviour following ART initiation is clearly an important area for future research, so that the risks and benefits of ART on HIV transmission can be more accurately estimated.

The potential risk of HIV transmission from this Medicare ineligible population has not been previously investigated. In the current era of treatment as prevention, consideration of this population needs to be included in any future policy. The NSW Ministry of Health HIV strategy 2012–2015 [10] has set a number of ambitious targets based on those agreed to under the 2011 United National Political Declaration on HIV and AIDS: Intensifying Our Efforts to Eliminate HIV and AIDS [11], which include working towards the virtual elimination of HIV and increased ART uptake to more than 90% of the HIV-positive population. Priority areas of action include the promotion of increased testing and treatment uptake and linking HIV-positive people to prevention, treatment and care services. For this “test and treat” approach to be successful in working towards the elimination of HIV, the HIV-positive temporary residents should be included. Similar goals to NSW are proposed in the QLD HIV Strategy 2013–2015 released in September 2013. It is expected that other states/territories may also endorse new HIV prevention and treatment targets in line with those endorsed by the Australian Health ministers [12].

In conclusion, doctors treating HIV-positive patients who are temporary residents and ineligible for healthcare under Medicare are having to manage these patients on limited resources, and are often unable to adequately fulfil their duty of care. Australian government policy allows these individuals to live and work in Australia, yet there remains a disconnect between Australian government policy in terms of the extent of support for these temporary residents compared with the current (and previous) National HIV Strategies [5,13]. The immunological and virological improvements highlight the importance of supplying ART to this population in need. The increase in the proportion with undetectable HIV viral load as early as six months demonstrates a potentially significant impact on the risk of onward HIV transmission in addition to the personal health benefit for each individual.

## Competing of interest

Bill Whittaker, Jo Watson, Lisa Bastian, Stephen T. Wright and Hamish McManus have no conflicts of interest. Kathy Petoumenos and Matthew Law's institution have received an unrestricted research grant from Gilead Science. Jennifer Hoy's institution has received funding for her participation in Advisory Boards for Merk, Sharp & Dohme, Gilead Sciences, ViiV Healthcare and Janssen-Cilag. Don Smith has received research grants from Gilead Sciences, Viiv Healthcare, MSD, Abbott Australasia, Kirby institute, NSW Department of Health, and educational support from Gilead Sciences, Viiv Healthcare, MSD, Abbott Australasia, BMS, NSW Department of Health. Robert Finlayson has received consultancy fees and educational grants from Abbott, BMS Boerhinger Ingelheim, Janssen Cilag, Gilead, MSD and ViiV.

## ATRAS sites

*New South Wales*: Albion Street Centre, Clinic 16, Royal North Shore, Holdsworth House Medical Practice, Darlinghurst, Nepean Sexual Health Clinic, Parramatta Sexual Health Clinic; RPA Sexual Health, St Vincent's Hospital, Sydney Sexual Health Clinic, Taylor Square Private Clinic, Northern Territory, Clinic 34.

*Queensland*: Brisbane Sexual Health and HIV Service, Cairns Sexual Health Clinic

Clinic 87.

*South Australia*: O'Brien Street Practice.

*Victoria*: Melbourne Sexual Health Clinic, Monash Medical Centre, Northside Clinic, Prahran Market Clinic, The Alfred Hospital.

*Western Australia*: Royal Perth Hospital

*Other*: Courtney Bendall, Adrian Ogier, Jae Condon, Lance Feeney.

## References

[1] NSW Health (on behalf of AHMAC) (2008). National study of medicare ineligible HIV positive temporary resident population in Australia.

[2] Department of Health and Ageing (2012). Pharmaceutical benefits schedule [Internet]. http://www.pbs.gov.au/medicine/item/9565K-9650X.

[3] Korner H (2007). “If I had my residency I wouldn't worry”: negotiating migration and HIV in Sydney, Australia. Ethn Health.

[4] Williams L, Foley S, Cain A (2011). Impact of medicare ineligibility on service delivery at Royal Perth Hospital. Australasian HIV/AIDS Conference.

[5] Commonwealth of Australia (2010). Sixth national HIV strategy 2010–2013.

[6] The Australian HIV Observational Database (2002). Rates of combination antiretroviral treatment change in Australia, 1997–2000. HIV Med.

[7] Wilson DP, Law MG, Grulich AE, Cooper DA, Kaldor JM (2008). Relation between HIV viral load and infectiousness: a model-based analysis. Lancet.

[8] Crepaz N, Hart TA, Marks G (2004). Highly active antiretroviral therapy and sexual risk behavior: a meta-analytic review. JAMA.

[9] The Kirby Institute (2013). HIV/AIDS, viral hepatitis and sexually transmissible infections in Australia Annual Surveillance Report.

[10] NSW Ministry of Health (2012). NSW HIV strategy 2012–2015: a new era.

[11] United Nations General Assembly (2011). Political declaration on HIV and AIDS: intensifying our efforts to eliminate HIV and AIDS. http://www.unaids.org/en/media/unaids/contentassets/documents/document/2011/06/20110610_UN_A-RES-65-277_en.pdf.

[12] Report and recommendations: progress progress on the Australian response to HIV and AIDS (Standing Council on Health 2013) [Internet]. https://www.health.gov.au/internet/main/publishing.nsf/Content/ohp-national-strategies-2010-hiv/$File/hiv.

[13] Commonwealth of Australia (2014). Seventh national HIV strategy 2014–2017.

